# Role of Endothelial Cell Metabolism in Normal and Tumor Vasculature

**DOI:** 10.3390/cancers15071921

**Published:** 2023-03-23

**Authors:** Alessio D’Alessio

**Affiliations:** Sezione di Istologia ed Embriologia, Dipartimento di Scienze della Vita e Sanità Pubblica, Università Cattolica del Sacro Cuore, 00168 Roma, Italy; alessio.dalessio@unicatt.it

Endothelial cells (ECs) form a simple squamous epithelium, the endothelium, which lines the lumen of all blood vessels and the heart. The endothelial layer, along with its underneath basal lamina, covered by a subendothelial connective tissue coating, forms the tunica intima, or interna, of all blood vessels. A thicker middle tunica media, mainly composed of varying amounts of elastic fibers and smooth muscle, and an outer tunica adventitia (or externa) of loose connective tissue account for the remaining covering structures of the vessel wall of the largest blood vessels [1] (Figure 1A). In contrast, the wall of capillaries, the smallest branches of the vascular tree that carry nutrients and oxygen to cells in our organs, is made exclusively from ECs and their basal lamina, covered by randomly scattered contractile pericytes [2] (Figure 1B).

In addition to forming a semi-permeable barrier that regulates exchanges of solutes between blood and the surrounding tissues across the blood vessel wall, healthy vascular ECs are dynamic and multifunctional cells that have countless functions in multiorgan health and homeostasis. These include the maintenance of vascular homeostasis [3], the formation of non-coagulant and non-thrombogenic surfaces [4,5], the control of blood flow and vascular tone, immune surveillance against foreign pathogens, leukocytes’ extravasation, and angiogenesis. The extraordinarily structural and functional high-phenotypic heterogeneity of endothelium has also been confirmed in single-cell sequencing studies [6,7]. Notably, vascular ECs are mostly dormant in adult vasculature [8,9], while they retain their capability of responding to external stresses and to restore their proliferative and migratory activity in response to angiogenic stimuli. However, this quiescent phenotype can be compromised by conditions that reduce or interfere with the vasoprotective homeostatic function of ECs, a condition referred to as endothelial dysfunction [10] (Figure 2).

In cancer, the presence of a largely disorganized blood vessel network corroborates the importance of the vascular endothelium as a major therapeutic target to counteract tumor development and progression. In this regard, while anti-angiogenic therapy, which aims to block Vascular Endothelial Growth Factor (VEGF) signaling, has received considerable attention in the field of oncology, it has proved to be inefficient in the treatment of certain cancers. This condition, which mainly results from the establishment of tumor resistance to treatment, and the activation of adaptive compensatory mechanisms [11,12,13] has prompted researchers to explore new strategies to counteract tumor angiogenesis. To this regard, it is becoming clear that the ability of vascular ECs to accomplish their multifaceted activities may rely on distinct metabolic pathways [14]. This brings us to the hypothesis that the identification of the exact mechanisms regulating the metabolic signature of ECs may increase our understanding of the molecular mechanisms underlying endothelial activation and dysfunction that occur in certain human pathologies, including atherosclerosis, diabetes, and cancer. 

This Special Issue of *Cancers*, dedicated to the “Role of Endothelial Cell Metabolism in Normal and Tumor Vasculature”, resulted in a total of six published articles, including one original research article and five review articles. In their research article, Tsai and collaborators investigated the role of bile acid signaling in pleural angiogenesis [15]. This study reveals a correlation between elevated bile acid levels and the increased expression of the bile acid farnesoid X receptor (FXR). The finding of an elevated expression level of FXR in Human Umbilical Vein Endothelial Cells treated with lung-cancer-associated pleural fluid (LCPF) is presented. The authors suggest that FXR modulation by statin reduces LCPF-induced angiogenesis and proliferation and plays a role in counteracting endothelial barrier disruption caused by LCPF. The review article by Testa and co-authors [16] recapitulates the dynamic interaction between brain microvasculature E (BMECs) and tumor cells and gives an overall description of how tumor cells take control of BMECs, promoting the formation of new blood vessels. First, they describe the normal behavior and functioning of BMECs in the brain, before discussing the features of glioblastoma (GBM)-associated BMECs. In addition, they dissect the multiple routes of BMEC–tumor cell communication. Specifically, they survey the dynamic and bi-directional interplay concerning the secretion or release of effector molecules such as VEGF, FGF, IL-8, miRNAs, long non-coding RNA (lnRNAs), and pro-angiogenic factors via extracellular vesicles (EVs). Lastly, the article discusses the putative advantages of new technical approaches to investigate cell–cell interactions, such as 3D tumor platforms. In the study entitled “Message in a bottle: Endothelial Cell Regulation by Extracellular Vesicles”, Palazzo and collaborators [17] present a detailed classification of the different types of EVs into subtypes, such as microvesicles, exosomes, apoptotic bodies, and oncosomes, based on their size and biogenesis. They deeply discuss the mechanisms by which EVs and their cargo regulate ECs’ phenotype, proliferation, migration, and tumor angiogenesis. The authors also emphasize the critical role of EVs in cell–cell communication, highlighting their fascinating role as potential therapeutic targets for the treatment of vascular dysfunction. In the review article entitled “Cancer-Induced Metabolic Rewiring of Tumor Endothelial Cells”, Lidonnici et al. [18] discuss the interaction of ECs with tumor cells. They focus on the mechanisms by which ECs rewire their metabolic status in cancers to readapt their metabolic needs, such as glycolysis, and the oxidation of fatty acid to the tumor environment. Their conclusions are in line with findings suggesting that metabolism-mediated angiogenesis is just as crucial as VEGF-mediated angiogenesis. The study stimulates future challenges aimed toward the investigation of how endothelial metabolism participates in vascular morphogenesis and differentiation, both in normal and pathological conditions. In their review article, Filippini et al. discuss the most recent evidence regarding the involvement of ECs’ metabolism in vascular functions [19]. The authors start by recapitulating studies describing both the embryological origin as well as the histological features of vascular endothelium. They report the most recent and relevant studies on the metabolic status role of ECs in several processes such as angiogenesis, endothelial activation, viral infection, and inflammation. Lastly, based on the high content of plasma membrane microdomains, such as lipid rafts and caveolae, found in ECs, the authors theorize on the as-yet poorly explored role of these structures on endothelial metabolism. The review article by Cutruzzolà [20] and colleagues discusses the intriguing and complex topic of cancer metastasis dissemination to the brain, focusing on the role of brain ECs in this process. The authors begin by revising the nature of the blood–brain barrier (BBB), the highly selective structure essential in safeguarding the central nervous system from harmful substances, thereby ensuring optimal neuronal function. They discuss the most recent findings concerning the role that amino acids such as glycine/serine and glutamate/glutamine have in the healthy brain and how these neurotransmitters induce cancer cells, with the complicity of brain ECs, to disseminate to brain tissues. Overall, the article underscores the importance of understanding the interplay between cancer cells and their microenvironment to develop effective strategies for preventing or treating cancer metastasis to the brain. In conclusion, I hope that this Special Issue of *Cancers* will be of interest to both scientists and physicians.

## Figures and Tables

**Figure 1:**
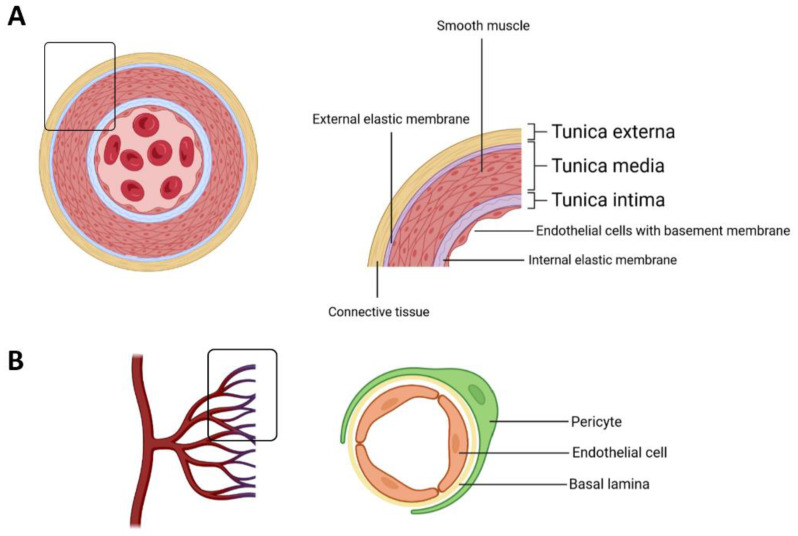
Schematic representation showing the main components of the blood vessel wall. (**A**) The transverse section of an artery; (**B**) cellular components of a blood capillary.

**Figure 2:**
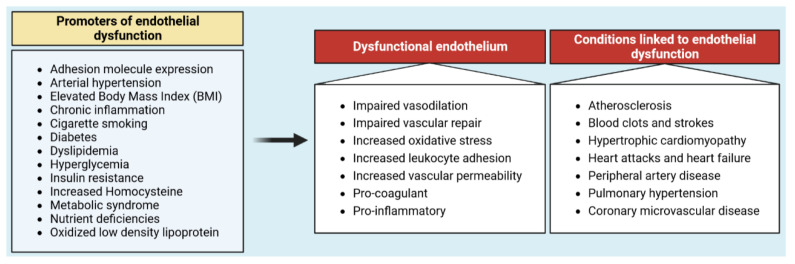
Major promoters of endothelial dysfunction and its associated conditions.
